# Comparative lobe-specific histomorphometric evaluation of pulmonary architecture, fibrosis, and alveolar macrophage distribution in swine raised under different management systems

**DOI:** 10.14202/vetworld.2026.422-439

**Published:** 2026-01-31

**Authors:** Nattawat Chaiyawong, Napat Praditwattanakit, Surachai Chamsodsai, Pichaya Jumnongprakhon, Ittipon Phoungpetchara, Charkriya Promsuban

**Affiliations:** 1 Siriraj Integrative Center for Neglected Parasitic Diseases, Department of Parasitology, Faculty of Medicine Siriraj Hospital, Mahidol University, Bangkok 10700, Thailand; 2 Faculty of Medical Science, Naresuan University, Phitsanulok, 65000, Thailand; 3 Department of Anatomy, Faculty of Medical Science, Naresuan University, Phitsanulok, 65000, Thailand; 4 Center of Excellence in Medical Biotechnology, Faculty of Medical Science, Naresuan University, Phitsanulok, 65000, Thailand

**Keywords:** alveolar macrophages, histomorphometry, pulmonary fibrosis, swine housing systems, lung remodeling, Masson’s trichrome staining

## Abstract

**Background and Aim:**

Swine housing and management systems strongly influence respiratory health through their effects on air quality, ventilation, and environmental exposure. However, quantitative, lobe-specific evidence describing how different management systems affect pulmonary microarchitecture remains limited. This study aimed to compare alveolar structure, fibrosis, collagen deposition, and alveolar macrophage distribution in swine raised under hygienic, beta-agonist-free rearing, and free-range systems.

**Materials and Methods:**

Fifteen clinically healthy male crossbred (Large White × Landrace) swine were allocated to three management systems (n = 5 per group): hygienic, beta-agonist-free rearing, and free-range. Lung samples were collected from the right cranial, middle, and caudal lobes following humane slaughter. Sections were stained with Masson’s trichrome for collagen visualization. Quantitative histomorphometric analyses included alveolar wall thickness, alveolar space area, fibrosis distribution (%), semi-quantitative fibrosis score, collagen intensity (mean gray value) in bronchial hyaline cartilage, and alveolar macrophage density (AMD). Image analysis was performed using ImageJ, and observers were blinded to group allocation. Data were analyzed using one-way analysis of variance with Tukey’s post hoc test (p < 0.05).

**Results:**

Marked lobe-specific differences were observed among management systems. Swine raised under beta-agonist-free rearing exhibited significantly thicker alveolar walls, reduced alveolar space area, higher fibrosis distribution and scores, increased collagen accumulation, and elevated AMD, particularly in the middle and caudal lobes. In contrast, free-range swine demonstrated thinner alveolar septa, wider alveolar spaces, lower fibrosis indices, and reduced macrophage infiltration, indicating preserved pulmonary architecture and reduced inflammatory remodeling. The hygienic group consistently showed intermediate values across most parameters, reflecting balanced structural adaptation under controlled housing conditions.

**Conclusion:**

Swine management systems are associated with distinct patterns of pulmonary structural and immunological adaptation. Beta-agonist-free rearing was linked to early fibrotic remodeling and increased immune activation, whereas free-range management supported structural preservation and lower inflammatory burden. This study provides the first quantitative, lobe-specific histomorphometric comparison of pulmonary remodeling across different swine management systems, offering valuable insights for welfare-oriented and sustainable livestock production strategies.

## INTRODUCTION

Swine farming systems play a decisive role in shaping physiological health, productivity, and overall welfare, as the management environment exerts profound effects on organ development, metabolism, and immune responses [1, 2]. The respiratory system is particularly sensitive to variations in environmental quality, stocking density, ventilation, and exposure to airborne contaminants such as dust, ammonia, and microbial aerosols [3, 4]. These factors influence not only respiratory efficiency but also the histological architecture of the lungs, which reflects the balance between tissue adaptation and chronic stress responses [5, 6]. Modern swine production encompasses several farming systems with differing priorities related to productivity, animal welfare, and food safety. Beta-agonist-free rearing, hygienic, and free-range systems represent distinct management approaches that differ in environmental control, dietary formulation, and biosecurity practices [7, 8]. To ensure residue-free pork and maintain metabolic stability, beta-agonist-free rearing systems eliminate pharmacological growth promoters [9]. Hygienic or intensive systems emphasize strict sanitation, controlled housing conditions, and veterinary-supervised health protocols to minimize pathogen exposure and optimize growth performance [10]. In contrast, free-range systems allow outdoor access and natural behaviors, conferring welfare benefits but increasing exposure to variable climatic conditions, environmental dust, and potential respiratory irritants [11, 12]. Despite these clear distinctions, the comparative effects of these management systems on swine pulmonary histology remain poorly characterized, representing a critical knowledge gap in understanding organ-specific adaptation to environmental and management conditions [13, 14]. The swine lung functions not only as a primary site for gas exchange but also as an active immunological organ that continuously interacts with environmental antigens [15, 16]. The bronchi, bronchioles, and alveoli are supported by a delicate connective tissue framework, within which the alveolar septa, containing capillaries and elastic fibers, are essential for maintaining surface area and elasticity during ventilation [17]. Structural alterations in these components may arise from oxidative stress, chronic inflammation, or exposure to airborne irritants, all of which are common under certain husbandry conditions [18, 19]. Excessive collagen deposition and interalveolar septal thickening are characteristic of pulmonary fibrosis (PF), a pathological process that compromises gas diffusion and lung compliance [20, 21]. Conversely, alveolar wall thinning and enlargement of alveolar spaces may reflect emphysematous or degenerative changes that reduce the effective surface area available for oxygen exchange [22]. Histopathological evaluation using Masson’s trichrome staining provides valuable insight into the extent and distribution of collagen deposition and fibrotic remodeling, as it clearly differentiates collagen from muscle and cytoplasmic elements [23]. In addition, quantitative assessment of alveolar wall thickness, alveolar space area, and alveolar macrophage density (AMD) provides an integrative perspective on pulmonary adaptation, tissue remodeling, and inflammatory status [24–26]. As the principal immune effector cells in the alveolar lumen, macrophages play a central role in particulate clearance, modulation of inflammation, and tissue repair [27]. Accordingly, variations in their abundance or morphology across farming systems may reflect differential immune challenges or adaptive responses to environmental conditions.

Although the effects of management systems on swine growth performance, carcass quality, and gastrointestinal histology have been widely documented [28–30], comparatively few studies have focused on pulmonary histology in swine raised under different housing and management regimes. This scarcity of comparative, quantitative, and lobe-specific pulmonary data represents a significant research gap, particularly given the role of the respiratory tract as both a functional organ and a sentinel indicator of environmental exposure and herd health [31].

Despite extensive documentation of the effects of different management systems on swine growth performance, carcass traits, and gastrointestinal histology [29, 30], evidence describing how these systems influence pulmonary microarchitecture remains limited. In particular, there is a lack of quantitative, lobe-specific data on how distinct rearing environments shape alveolar structure, fibrotic remodeling, and pulmonary immune cell distribution. Most available studies have relied on whole-lung or non-specific assessments, which may obscure region-dependent structural and immunological heterogeneity within the lung. Consequently, the relationships among housing conditions, environmental exposure, and micro-anatomical adaptations of the respiratory system remain poorly understood. This gap is especially important given that the lung serves not only as a gas exchange organ but also as a sensitive sentinel of environmental stress, inflammation, and long-term tissue remodeling. The absence of integrated histomorphometric analyses combining fibrosis, collagen deposition, and immune cell dynamics limits our ability to identify early or subclinical pulmonary adaptations associated with modern swine production systems.

Therefore, the present study was designed to quantitatively compare lobe-specific pulmonary histology, fibrosis, and alveolar macrophage distribution in swine raised under beta-agonist-free, hygienic, and free-range rearing systems. We hypothesized that beta-agonist-free rearing and free-range management would exhibit distinct patterns of alveolar remodeling and immune cell distribution, reflecting differential physiological adaptation to environmental and management conditions. To test this hypothesis, collagen accumulation, fibrosis severity, alveolar wall thickness, alveolar space area, and alveolar macrophage distribution were systematically evaluated using Masson’s trichrome staining and standardized histomorphometric analysis. By integrating structural remodeling, extracellular matrix (ECM) deposition, and immune cell dynamics at a micro-anatomical and lobe-specific level, this study provides one of the first comprehensive assessments of pulmonary adaptation across contrasting swine management systems. The findings are expected to advance understanding of how farming environments influence respiratory tissue health and microstructural integrity in swine, thereby offering a histological basis for the development of welfare-oriented and sustainable livestock management practices.

## MATERIALS AND METHODS

### Ethical approval

All experimental procedures involving animals were conducted in accordance with the guidelines for the care and use of laboratory animals of the National Institutes of Health. Ethical approval was granted by the Animal Ethics Committee, Center for Animal Research, Naresuan University (Approval No. NU-AEE620505) before the initiation of the study. All procedures, including housing, management, and tissue collection, were performed in accordance with the Animal Research: Reporting of *In Vivo* Experiments 2.0 guidelines to ensure ethical treatment, animal welfare, and transparent reporting.

### Study period and location

The study was conducted from October 2024 to October 2025 in Phitsanulok, Thailand. Lung samples were collected from swine raised under different management systems, including intensive (hygienic), beta-agonist-free, and free-range systems. Animals were obtained from commercial farms and/or local production systems representative of each management condition. Histological processing, staining, and lobe-specific histomor-phometric analyses were performed at the Department of Anatomy Laboratory, Naresuan University, Thailand.

### Animal selection and grouping

A total of 15 clinically healthy male crossbred (Large White × Landrace) swine, aged 5–6 months and weighing 90–110 kg, were included in this study. Animals were sourced from representative farms operating under distinct management systems within the same geographic region of Thailand. Swine were allocated to three groups according to farming system: beta-agonist-free rearing, hygienic, and free-range. Each group comprised five animals derived from farms with consistent management practices specific to each system and exposed to comparable regional climatic conditions. All animals were maintained continuously under their respective management systems from the post-weaning period until slaughter (~5–6 months) to allow adequate physiological adaptation.

### Housing and management conditions

In the beta-agonist-free rearing system, animals were housed in conventional facilities at a stocking density of approximately 0.8–1.0 animals/m², with natural ventilation and routine cleaning schedules. In the hygienic system, animals were maintained in controlled indoor environments with established biosecurity protocols, regular veterinary supervision, and commercial feed formulated for optimal growth (18%–20% crude protein, 3.3–3.5 Mcal/kg metabolizable energy, 3%–5% fiber). Temperature (25°C–28°C) and humidity (60%–70%) were maintained within recommended ranges, with a stocking density of 1.0–1.2 animals/m² and mechanical ventilation providing 6–8 air changes per hour. In the free-range system, animals were reared outdoors with access to soil and natural vegetation and were fed a combination of commercial and locally available feed sources with ad libitum access and seasonal forage supplementation. Stocking density was approximately 0.5 animals/m², and natural ventilation was used. Although temperature and humidity were not strictly controlled, climatic conditions were comparable among groups due to the shared geographic region and season. All animals had ad libitum access to water, and farm management characteristics, including housing type, feeding strategy, health monitoring, and veterinary interventions, were documented to minimize farm-level confounding.

### Tissue collection and humane slaughter

Tissue collection followed standard commercial swine slaughter procedures under veterinary supervision. Animals were transported to the processing facility, allowed an appropriate resting period, and slaughtered humanely using electrical stunning followed by exsanguination, in compliance with institutional and national animal care and welfare guidelines, including those of the Thai Department of Livestock Development. Stunning parameters were applied to minimize distress and ensure immediate loss of consciousness before bleeding.

### Lung lobe selection

Following humane slaughter, lung samples were immediately collected from the right cranial, middle, and caudal lobes of each animal. The right lung was selected because of its larger size and distinct lobulation, which enabled reproducible region-specific histomorphometric comparisons. Sampling a single lung side minimized anatomical variability while capturing representative regions of ventilation, perfusion, and mechanical loading, thereby allowing standardized assessment of pulmonary structure across animals and management systems.

### Randomization and field selection

Animals were randomly selected from representative farms within each management system. For each lung lobe, microscopic fields for histomorphometric analysis were randomly chosen to minimize selection bias and ensure representative sampling.

### Tissue processing

Tissue specimens measuring approximately 1 × 1 × 0.5 cm were excised and fixed in 10% neutral buffered formalin at room temperature for 7 days. Following fixation, tissues were processed using graded alcohols and xylene, embedded in paraffin wax, and sectioned at a thickness of 5 µm with a rotary microtome. Sections were mounted on poly-L-lysine-coated glass slides and air-dried before staining.

### Masson’s trichrome staining

Masson’s trichrome staining was performed according to standard histological protocols for the detection and differentiation of collagen fibers [32]. Briefly, paraffin-embedded sections were deparaffinized in xylene, rehydrated through graded ethanol, and sequentially stained with Weigert’s iron hematoxylin for nuclei, Biebrich scarlet–acid fuchsin for cytoplasm and muscle fibers, and aniline blue for collagen fibers. Differentiation was achieved using phosphomolybdic–phosphotungstic acid, followed by dehydration, clearing, and mounting with a resinous medium. Collagen fibers appeared blue, whereas muscle and cytoplasmic elements stained red.

### Image acquisition and calibration

Quantitative analyses were conducted using a light microscope equipped with a digital imaging system. Images were captured at 100× and 400× magnification under consistent illumination and calibrated scale. All images were analyzed using standardized threshold settings in ImageJ (version 1.54p; National Institutes of Health, USA). Calibration was performed using a stage micrometer to convert pixels to micrometers, and identical parameters were applied uniformly across all samples to ensure reproducibility and minimize analytical bias.

### Alveolar wall thickness and alveolar space measurement

Alveolar wall thickness was determined as the mean thickness of 10 randomly selected alveolar septa per microscopic field, with at least five non-overlapping fields analyzed per lobe. At least one section per lobe was examined for each animal, and field selection was randomized. Measurements were performed using the straight-line tool on calibrated images (µm/pixel). Alveolar space area was quantified by threshold adjustment to delineate alveolar lumens, followed by particle analysis in ImageJ to calculate airspace area as a percentage of total parenchymal area. Regions containing large bronchioles or blood vessels were excluded to avoid structural bias.

### Fibrosis assessment

Quantitative assessment of fibrosis distribution (%) was performed using ImageJ software. Digital images were converted to grayscale, and collagen was isolated using the color deconvolution (Masson’s trichrome) plugin. Threshold settings were standardized across all images. The proportion of fibrotic area relative to total parenchymal area was calculated using the analyze particles function. Semi-quantitative fibrosis scoring followed the grading system proposed by Hübner *et al*. [33], ranging from 0 (normal lung architecture, no fibrosis) to 8 (complete fibrotic obliteration). Scores reflected the extent and severity of collagen deposition and architectural distortion: 0–1, normal or minimal fibrotic thickening; 2–4, moderate collagen deposition with focal interstitial expansion and partial alveolar collapse; 5–8, extensive fibrosis with marked structural distortion and loss of normal alveolar spaces [34]. Mean fibrosis scores were calculated per animal from all observed fields. Inter-observer agreement was evaluated using weighted kappa statistics (κ = 0.78), indicating substantial concordance, and final scores represented the average of three blinded observers.

### Collagen intensity in bronchial cartilage

Collagen intensity within bronchial hyaline cartilage (BHC) was quantified using the mean gray value method. Regions of interest were delineated along hyaline cartilage plates, excluding peribronchial connective tissue and smooth muscle. Blue-stained collagen areas were analyzed in ImageJ following color deconvolution. Lower mean gray values indicated stronger blue staining and higher collagen density. At least three regions of interest per section were analyzed, with measurements independently performed by three blinded observers and consistency assessed using intraclass correlation coefficients.

### Quantification of alveolar macrophages

Alveolar macrophages were identified morphologically as round-to-oval cells with dense basophilic nuclei, pale cytoplasm, and typical localization within alveolar lumens adjacent to septa. Lymphocytes, neutrophils, and other mononuclear cells were excluded. A minimum of five randomly selected non-overlapping fields per sample was analyzed to ensure representative and unbiased quantification.

### Blinding and standardization

All histological evaluations were conducted on coded slides, and observers were blinded to management system and lung lobe identity. Field selection, section number, region-of-interest delineation, exclusion criteria, and calibration parameters were standardized across all samples to ensure methodological consistency and reproducibility.

### Statistical analysis

#### Data presentation and normality testing

All quantitative data are presented as mean ± standard error of the mean. Data normality was assessed using the Shapiro–Wilk test, and homogeneity of variances was evaluated using Levene’s test.

#### Statistical comparisons among groups

Comparisons among the three farming systems were performed using one-way analysis of variance (ANOVA), followed by Tukey’s post hoc test for multiple comparisons. Tukey’s test was applied to control the family-wise error rate and reduce the risk of type I error.

#### Nested data structure and experimental units

Multiple fields per lobe were averaged to account for the nested data structure and generate a single value per lobe per animal. Each animal was treated as the experimental unit to ensure statistical independence and minimize intra-animal clustering. Data from different lung lobes were analyzed separately to allow region-specific comparisons.

#### Reporting of significance and software

Exact p-values are reported for all statistical tests, and statistical significance was defined as p < 0.05. All analyses were performed using GraphPad Prism version 10.6.1. Mixed-effects models were evaluated; however, separate lobe-level analyses with animals as the experimental units were considered appropriate given the study design and sample size.

## RESULTS

### Alveolar wall thickness across lung lobes

**Figure 1:**
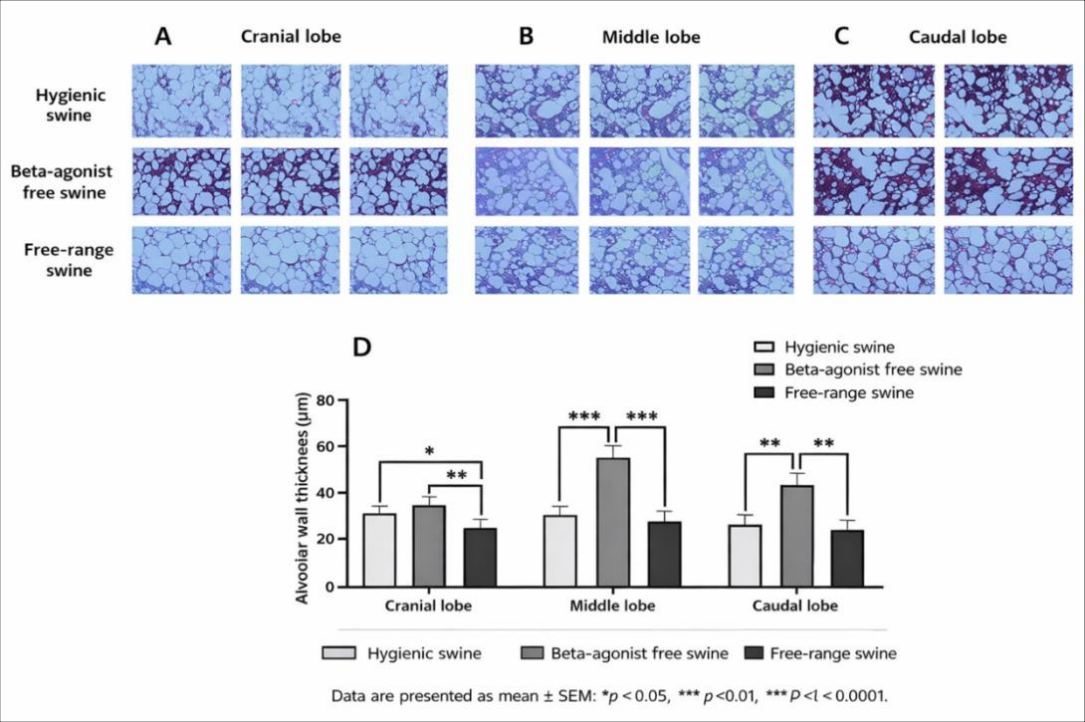
(A) Cranial, (B) middle, and (C) caudal lung lobes showing representative Masson’s trichrome-stained sections from hygienic, beta-agonist-free, and free-range pigs. (D) Quantitative comparison of alveolar wall thickness (µm) among the experimental groups. Data are presented as mean ± standard error of the mean; *p < 0.05, **p < 0.01, ***p < 0.0001.

Mean alveolar wall thickness differed significantly among the three farming systems across all lung lobes. In the cranial lobe, the hygienic group exhibited an average alveolar wall thickness of 31.67 ± 2.94 µm, whereas the beta-agonist-free rearing group showed 33.40 ± 2.94 µm, and the free-range group demonstrated 24.60 ± 2.94 µm. Statistical analysis revealed significant differences between the hygienic and free-range groups (p = 0.0463) and between the beta-agonist-free rearing and free-range groups (p = 0.0054) (Figures 1A and 1D).

In the middle lobe, alveolar wall thickness was markedly greater in the beta-agonist-free rearing group (56.57 ± 4.30 µm) than in both the hygienic (29.51 ± 4.30 µm) and free-range (27.16 ± 4.30 µm) groups, with highly significant differences observed for both comparisons (p = 0.0000) (Figures 1B and 1D). A similar pattern was evident in the caudal lobe, where alveolar walls were thicker in the beta-agonist-free rearing group (46.39 ± 3.32 µm) compared with the hygienic (26.73 ± 3.32 µm) and free-range (25.09 ± 3.32 µm) groups, again demonstrating strong statistical significance (p = 0.0000 for both comparisons) (Figures 1C and 1D).

Overall, the beta-agonist-free rearing group consistently exhibited the thickest alveolar septa, particularly in the middle and caudal lobes, whereas the free-range group displayed the thinnest alveolar walls across all examined regions. A post hoc power analysis based on the observed differences in alveolar wall thickness was conducted using a one-way ANOVA model with an alpha level of 0.05. The large effect sizes observed in the middle and caudal lung lobes (η² > 0.60) resulted in statistical power exceeding 0.90 with five animals per group. Although the cranial lobe demonstrated a smaller effect size, the achieved power remained acceptable (>0.80) for detecting differences between the free-range and other management systems. These findings indicate that the sample size was sufficient to detect biologically meaningful differences in alveolar wall thickness among groups.

### Alveolar space area and airspace configuration

The presence of thicker alveolar walls suggests potential interstitial expansion or fibrotic remodeling, which may influence overall alveolar architecture. In line with these structural changes, analysis of the alveolar space area revealed complementary differences among groups, reflecting the impact of septal thickness on functional airspace and gas exchange capacity.

**Figure 2:**
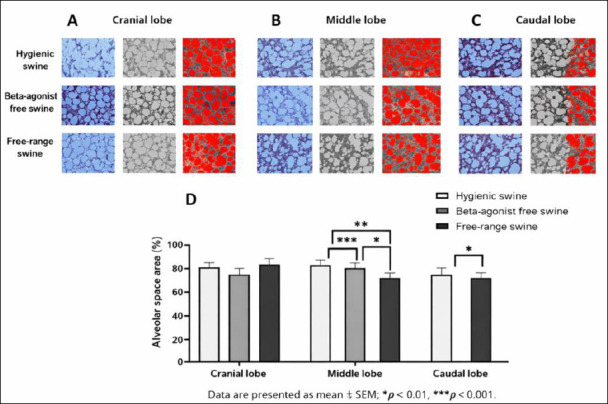
(A) Cranial, (B) middle, and (C) caudal lung lobes showing representative Masson’s trichrome-stained sections from hygienic, beta-agonist-free, and free-range pigs. (D) Quantitative comparison of alveolar space area (%), with higher values indicating larger alveolar lumens, across the experimental groups. Data are presented as mean ± standard error of the mean; *p < 0.05, **p < 0.01, ***p < 0.0001.

Quantitative assessment demonstrated clear differences in alveolar space area among the three management systems across all pulmonary lobes. In the cranial lobe, alveolar space area averaged 80.26% ± 4.78% in the hygienic group, 75.57% ± 3.48% in the beta-agonist-free rearing group, and 85.04% ± 3.93% in the free-range group (Figure 2A and 2D).

In the middle lobe, the hygienic group exhibited the largest alveolar spaces (93.39% ± 3.36%), followed by the free-range (79.71% ± 3.27%) and beta-agonist-free rearing (71.08% ± 4.30%) groups. Statistical analysis showed highly significant differences between the hygienic and beta-agonist-free rearing groups (p = 0.0000), between the hygienic and free-range groups (p = 0.0013), and between the beta-agonist-free rearing and free-range groups (p = 0.0356) (Figures 2B and 2D).

In the caudal lobe, alveolar space area was 80.26% ± 4.78% in the hygienic group, 75.57% ± 3.48% in the beta-agonist-free rearing group, and 85.04% ± 3.93% in the free-range group, with a statistically significant difference detected between the beta-agonist-free rearing and free-range groups (p = 0.0266) (Figures 2C and 2D).

Overall, the beta-agonist-free rearing group consistently exhibited the smallest alveolar spaces, whereas the free-range group tended to show the largest, indicating that management system and environmental exposure strongly influenced alveolar structural configuration. Post hoc power analysis using one-way ANOVA (α = 0.05) revealed large effect sizes in the middle and caudal lung lobes (η² > 0.50), yielding statistical power >0.85 with five animals per group. Although the cranial lobe showed a smaller effect size, power remained acceptable (>0.80), confirming an adequate sample size to detect biologically meaningful differences in alveolar space area.

### PF distribution and severity

Differences in alveolar space area among groups reflect variations in parenchymal expansion and ventilation and suggest underlying ECM remodeling. These structural alterations were therefore further examined through quantitative and semi-quantitative assessment of PF to relate alveolar geometry to fibrotic deposition and tissue remodeling patterns.

**Figure 3:**
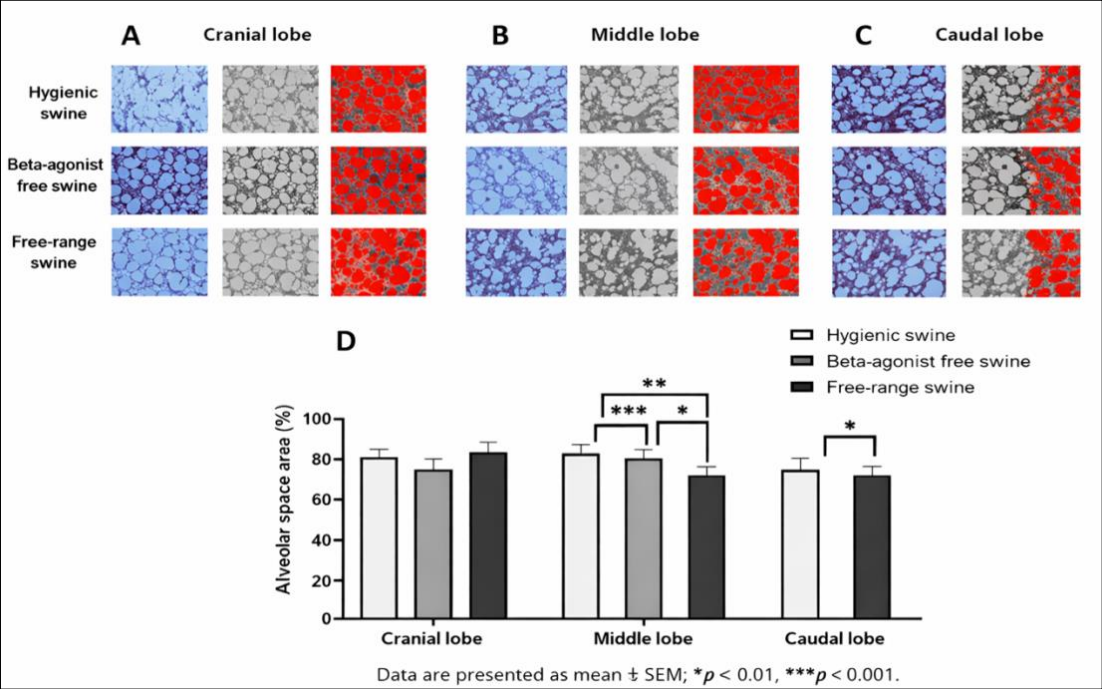
(A) Cranial, (B) middle, and (C) caudal lung lobes showing representative Masson’s trichrome-stained sections from swine reared under hygienic, beta-agonist-free, and free-range systems, highlighting collagen-rich fibrotic regions. (D) Quantitative comparison of fibrosis distribution (%) and (E) fibrosis scores (0–8) across the experimental groups, with higher values indicating greater collagen accumulation. Data are presented as mean ± standard error of the mean; **p < 0.01, ***p < 0.0001.

Quantitative analysis revealed distinct differences in both fibrosis distribution and fibrosis score among management systems across all lung lobes. In the cranial lobe, fibrosis distribution averaged 11.75% ± 1.20% in the hygienic group, 14.30% ± 1.50% in the beta-agonist-free rearing group, and 9.20% ± 1.10% in the free-range group, corresponding to fibrosis scores of 1.17 ± 0.30, 1.50 ± 0.30, and 0.83 ± 0.30, respectively. Significant differences were observed in fibrosis distribution among all group comparisons (p = 0.0023–0.0000); however, fibrosis scores did not differ significantly among groups in this lobe (Figure 3A, 3D, and 3E).

In the middle lobe, fibrosis distribution was 18.00% ± 2.00% in the hygienic group, 28.50% ± 2.00% in the beta-agonist-free rearing group, and 21.50% ± 1.50% in the free-range group, with corresponding fibrosis scores of 2.00 ± 0.41, 4.50 ± 0.41, and 2.50 ± 0.41, respectively. Differences in both fibrosis distribution (p = 0.0000–0.0058) and fibrosis scores (p = 0.0021–0.0065) were statistically significant (Figures 3B, 3D, and 3E).

In the caudal lobe, fibrosis distribution was 14.30% ± 1.50% in the hygienic group, 25.50% ± 2.00% in the beta-agonist-free rearing group, and 14.30% ± 1.20% in the free-range group, with fibrosis scores of 1.50 ± 0.40, 3.50 ± 0.40, and 1.50 ± 0.40, respectively. Both fibrosis distribution and fibrosis scores were significantly higher in the beta-agonist-free rearing group compared with the hygienic and free-range groups (p = 0.0000–0.0065) (Figures 3C, 3D, and 3E).

Overall, the beta-agonist-free rearing group consistently exhibited the highest fibrosis distribution and fibrosis scores across all lung lobes, particularly in the middle and caudal regions. In contrast, the free-range group demonstrated the lowest levels of fibrotic change, while the hygienic group showed moderate fibrotic involvement, indicating partial interstitial remodeling with preserved alveolar architecture. Post hoc power analysis confirmed adequate sample size to detect pronounced fibrotic remodeling (η² > 0.60, power >0.90), with lower power in the cranial lobe corresponding to smaller effect sizes.

### Collagen intensity in BHC

**Figure 4:**
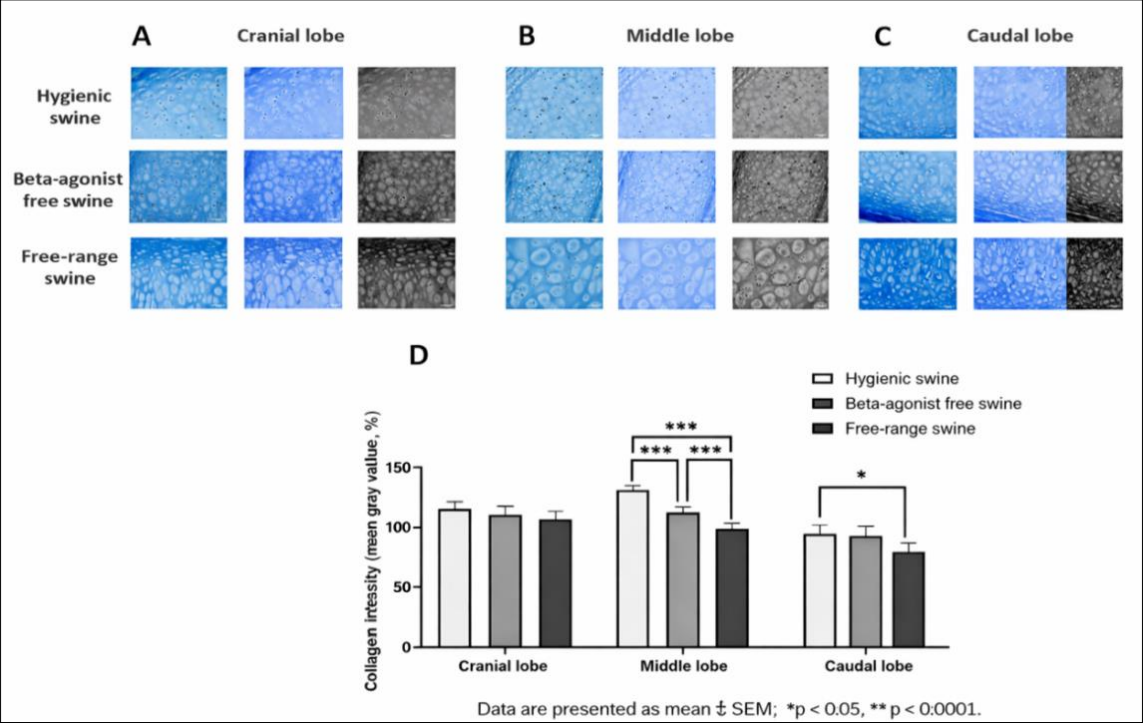
(A) Cranial, (B) middle, and (C) caudal lung lobes showing representative Masson’s trichrome-stained sections illustrating collagen distribution in the bronchial hyaline cartilage of swine reared under hygienic, beta-agonist-free, and free-range systems. (D) Quantitative comparison of collagen intensity (mean gray value) across the experimental groups, with lower values indicating denser collagen. Data are presented as mean ± standard error of the mean; *p < 0.05, ***p < 0.0001.

The observed differences in PF provided a macroscopic indication of fibrotic remodeling, which was further explored at the micro-anatomical level by quantifying collagen intensity (mean gray value) in BHC. This analysis enabled correlation between overall fibrotic deposition and localized collagen accumulation within the bronchial framework. In the cranial lobe, mean gray values were 119.8 ± 5.55 in the hygienic group, 110.8 ± 5.55 in the beta-agonist-free rearing group, and 108.7 ± 5.55 in the free-range group. Although these differences were not statistically significant, a trend toward higher mean gray values, indicating lower collagen deposition, was observed in the hygienic group (Figure 4A and 4D).

In the middle lobe, mean gray values were 134.3 ± 3.11, 111.1 ± 3.11, and 95.6 ± 3.11 in the hygienic, beta-agonist-free rearing, and free-range groups, respectively, with highly significant differences among all pairwise comparisons (p = 0.0000) (Figure 4B and 4D). In the caudal lobe, mean gray values were 111.1 ± 8.5 in the hygienic group, 102.0 ± 8.5 in the beta-agonist-free rearing group, and 91.0 ± 8.5 in the free-range group, with a significant difference detected between the hygienic and free-range groups (p = 0.0210) (Figures 4C and 4D).

Overall, the free-range group exhibited the lowest mean gray values across all lobes, indicating increased collagen deposition within BHC, whereas the hygienic group showed the highest mean gray values, reflecting reduced collagen accumulation. The beta-agonist-free rearing group displayed intermediate collagen intensity, suggesting variable remodeling responses. Power analysis demonstrated high power (>0.95) in the middle lobe and moderate power in the cranial and caudal lobes.

### AMD

To further investigate the relationship between structural remodeling and immune response, AMD was assessed following evaluation of collagen intensity in BHC. This approach allowed examination of whether increased collagen deposition corresponded to alterations in local immune cell populations.

**Figure 5:**
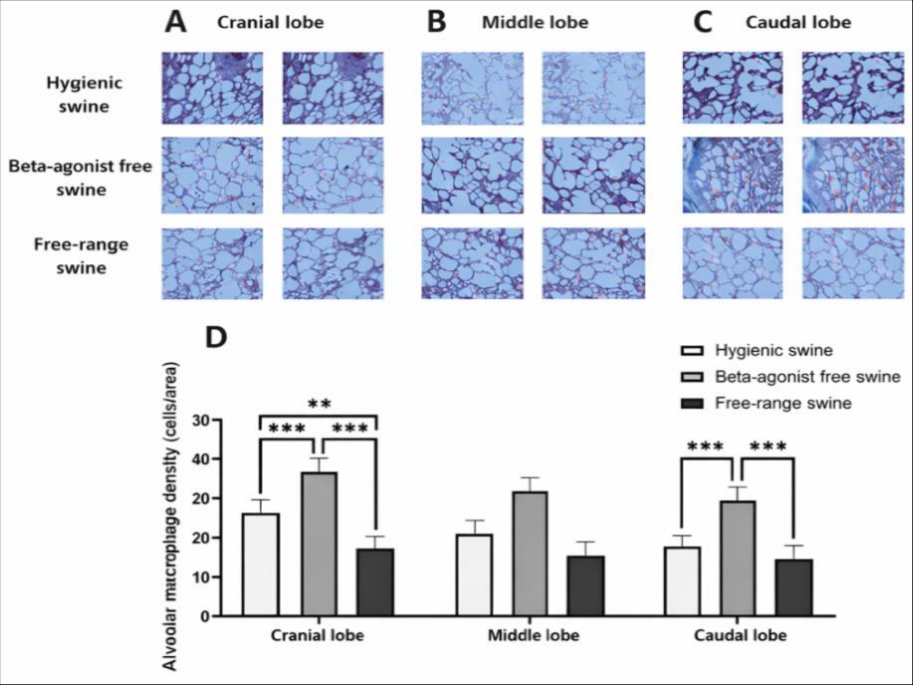
(A) Cranial, (B) middle, and (C) caudal lung lobes showing representative Masson’s trichrome-stained sections illustrating alveolar macrophages (red circles) in hygienic, beta-agonist-free, and free-range pigs. (D) Quantitative comparison of alveolar macrophage density (cells/unit area) across the experimental groups, with higher values indicating increased immune activity. Data are presented as mean ± standard error of the mean; **p < 0.01, ***p < 0.0001.

In the cranial lobe, AMD averaged 18.22 ± 2.22 cells/area in the hygienic group, 28.11 ± 2.38 cells/area in the beta-agonist-free rearing group, and 9.71 ± 3.27 cells/area in the free-range group, with significant differences observed among all comparisons (p = 0.0046–0.0000) (Figures 5A and 5D).

In the middle lobe, AMD values were 13.56 ± 2.92, 19.56 ± 3.01, and 13.63 ± 3.01 cells/area in the hygienic, beta-agonist-free rearing, and free-range groups, respectively, showing a trend toward higher macrophage density in the beta-agonist-free rearing group, although the difference did not reach statistical significance (Figures 5B and 5D).

In the caudal lobe, AMD was significantly higher in the beta-agonist-free rearing group (22.33 ± 2.46 cells/area) than in the hygienic (12.27 ± 2.26 cells/area) and free-range (9.33 ± 1.97 cells/area) groups (p = 0.0004–0.0000) (Figures 5C and 5D).

Overall, the beta-agonist-free rearing group consistently exhibited the highest AMD across all lobes, whereas the free-range group showed the lowest, with the hygienic group presenting intermediate values. Post hoc power analysis using one-way ANOVA (α = 0.05) indicated large effect sizes in the cranial and caudal lobes (η² > 0.55) and statistical power exceeding 0.90, confirming that the sample size was adequate to detect biologically relevant differences in macrophage infiltration.

## DISCUSSION

### Alveolar wall thickness and pulmonary structural integrity

Alveolar wall thickness is a key indicator of pulmonary integrity and gas exchange efficiency [35]. Under physiological conditions, thin alveolar septa facilitate efficient oxygen diffusion between alveolar air and capillary blood, whereas thickening of the alveolar walls reflects fibrotic remodeling, edema, or chronic inflammation that can impair gas exchange and reduce pulmonary compliance [36, 37]. In the present study, swine raised under the beta-agonist-free rearing system exhibited significantly increased alveolar wall thickness compared with the other groups. This observation indicates a previously underappreciated pulmonary trade-off associated with beta-agonist-free rearing: although pharmacological growth promoters are avoided, animals may undergo early fibrotic and low-grade inflammatory remodeling as a consequence of conventional indoor housing conditions [38]. These findings suggest an early tissue-level adaptation to management-associated environmental stressors, integrating structural remodeling with subtle inflammatory and immune modulation.

The absence of beta-agonists may influence pulmonary hemodynamics and smooth muscle tone, potentially triggering subtle inflammatory or fibrotic alterations within the lung parenchyma [39]. Collectively, these observations indicate that the beta-agonist-free system, despite being considered a neutral management alternative, imposes specific physiological costs on lung tissue, reflecting a complex interplay among housing conditions, nutrition, and pulmonary adaptation. Furthermore, the observed septal thickening likely reflects the combined effects of feed composition, indoor air quality, ventilation efficiency, and other farm-level factors, underscoring the multifactorial nature of pulmonary remodeling under conventional rearing conditions [40].

In contrast, the free-range group exhibited thinner alveolar walls, suggesting improved pulmonary elasticity and ventilation efficiency. Outdoor housing provides superior air circulation and promotes natural physical activity, thereby enhancing alveolar expansion and oxygen exchange [41]. Structural preservation in this system may represent an adaptive phenotype in which mechanical stretch, natural ventilation, and reduced exposure to indoor particulates synergistically maintain alveolar microarchitecture and limit fibrotic deposition. Nevertheless, the combination of thinner septa and larger alveolar spaces may also indicate early alveolar simplification or mild emphysematous change [42]. The hygienic group demonstrated intermediate values, reflecting a balance between controlled housing and environmental exposure [43]. This pattern supports the concept that optimal environmental control can preserve pulmonary structural integrity while providing limited physiological stimulation, thereby minimizing both excessive fibrosis and alveolar simplification.

Collectively, these findings demonstrate that the management environment exerts a measurable influence on lung microarchitecture, with alveolar wall thickness representing an integrated outcome of structural, immunological, and environmental interactions. Specifically, beta-agonist-free rearing is associated with early fibrotic adaptation and increased immune activation, free-range management promotes alveolar expansion and functional resilience, and hygienic systems maintain structural equilibrium, illustrating organ-specific adaptation to distinct husbandry practices.

### Alveolar space area and functional gas exchange

Alveolar space area provides a complementary index of parenchymal expansion and functional gas exchange capacity [44]. The reduced alveolar space observed in the beta-agonist-free rearing group is consistent with the presence of thicker alveolar septa, suggesting alveolar compression or interstitial matrix deposition resulting from inflammatory infiltration or early fibrotic remodeling [45]. This restrictive parenchymal pattern may compromise ventilation efficiency and impair oxygen diffusion [46].

In contrast, the free-range group exhibited significantly wider alveolar spaces, reflecting enhanced alveolar recruitment and improved air exchange. Increased physical activity and outdoor exposure likely promoted alveolar expansion and prevented atelectasis in this group [47]. Although these changes are largely indicative of adaptive remodeling, the possibility of overdistension and mild emphysematous-like alterations cannot be completely excluded, particularly if such conditions persist over extended periods [48, 49]. The hygienic group again displayed intermediate characteristics, maintaining relatively large alveolar areas, especially in the middle lobe, without evidence of overexpansion [50]. The controlled, biosecure environment associated with hygienic housing, combined with adequate ventilation and low particulate exposure, appears to support efficient oxygen exchange while minimizing structural stress [51].

Overall, swine raised under beta-agonist-free rearing conditions exhibited a restrictive pulmonary remo-deling pattern characterized by increased alveolar wall thickness and reduced alveolar space. From a histo-physiological perspective, these concurrent alterations reflect an integrated inflammatory–fibrotic response within the alveolar microenvironment. Conversely, free-range rearing supported structural preservation, as indicated by thinner alveolar walls and wider alveolar spaces, consistent with enhanced ventilation, physical activity, and adaptation to natural environmental conditions. The hygienic system demonstrated intermediate features, suggesting that pulmonary structural integrity can be maintained in controlled housing with adequate ventilation without inducing excessive remodeling. These observations highlight the direct influence of management conditions on pulmonary architecture and ventilation efficiency in swine.

### PF and collagen deposition

The increased fibrosis observed in the beta-agonist-free rearing group underscores a previously under-recognized pulmonary trade-off associated with this management system, in which avoidance of pharmacological growth promoters may inadvertently predispose swine to early interstitial remodeling and chronic low-grade inflammatory stress. Elevated collagen accumulation and fibroblast activation are hallmarks of early fibrotic change and may arise from persistent low-grade inflammation or mechanical stress within the lung parenchyma [52]. Management-related factors, including indoor housing, ventilation limitations, and feed composition, may act synergistically to alter airway smooth muscle tone and pulmonary vascular flow, thereby promoting ECM deposition [53]. The concomitant thickening of alveolar walls and reduction in alveolar space observed in this group is consistent with a restrictive lung phenotype characteristic of fibrotic adaptation [54].

By contrast, the free-range group exhibited minimal fibrotic involvement, indicating preservation of normal parenchymal integrity. Under free-range conditions, regular exercise, improved air quality, and natural ventilation are likely to reduce oxidative stress and tissue hypoxia—both strong drivers of fibroblast activation [55]. As a result, collagen accumulation was limited, consistent with healthy tissue remodeling [56]. The hygienic group displayed moderate fibrosis, suggesting a balance between controlled management and limited environmental stress [57, 58]. Spatially, fibrotic changes were more pronounced in the middle and caudal lobes across all groups, reflecting regional differences in mechanical load and ventilation–perfusion gradients that predispose these areas to remodeling [34, 59]. These findings confirm that management systems influence not only the extent but also the regional distribution of PF in swine.

It should be noted that fibrosis assessment in the present study relied primarily on Masson’s trichrome staining and semi-quantitative histological scoring, without molecular or immunohistochemical validation of fibrotic pathways such as collagen subtypes, α-smooth muscle actin, or TGF-β signaling. Accordingly, the observed alterations reflect structural remodeling rather than definitive molecular activation of fibrosis pathways. Future investigations incorporating complementary molecular or immunohistochemical approaches are warranted to clarify mechanistic links between management environment and production/farrowing rate in swine.

### Bronchial cartilage collagen remodeling

Collagen deposition within bronchial cartilage also varied among management systems and appeared to reflect distinct adaptive mechanisms. Notably, increased bronchial cartilage collagen was observed in the free-range group, despite minimal parenchymal fibrosis and low AMD. This unexpected finding suggests that outdoor conditions, mechanical demands, and environmental exposure may drive localized connective tissue remodeling even under relatively low inflammatory stress. Such divergent responses between alveolar parenchyma and bronchial cartilage highlight the complexity of pulmonary adaptation and demonstrate that structural remodeling is not uniformly attenuated under free-range management.

The elevated collagen intensity observed in the free-range group may represent a compensatory response to increased mechanical ventilation demands, temperature fluctuations, and environmental stressors [60]. Structural remodeling of the bronchial wall may therefore function as an adaptive mechanism to maintain airway stability under dynamic respiratory loading in variable outdoor environments. Enhanced collagen synthesis within the bronchial wall likely contributes to airway stabilization during repeated mechanical strain [61]. Repetitive mechanical stress and oxidative stimuli can upregulate fibroblast activity, resulting in localized collagen accumulation as part of a protective remodeling process [62]. However, excessive collagen deposition may ultimately reduce tissue elasticity and compromise pulmonary compliance.

In contrast, the hygienic group exhibited the lowest collagen intensity, consistent with a controlled and low-stress housing environment [63]. Limited structural remodeling in this group underscores the organ-specific capacity for adaptive ECM regulation under stable environmental conditions. Controlled temperature, humidity, and air quality reduce the need for structural adaptation, thereby maintaining balanced ECM turnover and tissue elasticity [64]. Intermediate collagen levels in the beta-agonist-free rearing group may reflect altered metabolic regulation following the removal of beta-agonist compounds, which are known to influence smooth muscle tone and connective tissue metabolism [65]. Collectively, these patterns illustrate that bronchial collagen deposition arises from coordinated interactions among mechanical, environmental, and metabolic factors, highlighting the adaptive plasticity of airway connective tissue across management systems. Overall, these findings indicate that housing conditions affect not only alveolar parenchyma but also the connective tissue framework of the bronchial wall [66]. Continuous assessment of collagen remodeling using histomorphometric indices, such as mean gray value, may serve as a useful biomarker for evaluating pulmonary adaptation and welfare across different management systems [67, 68].

### AMD and immune response

AMD provides additional insight into the immunological mechanisms underlying pulmonary adaptation. Macrophages are central to lung homeostasis through their roles in phagocytosis, cytokine production, and modulation of inflammatory processes [69]. The significantly elevated macrophage density observed in the beta-agonist-free rearing group indicates persistent immune activation and chronic low-grade inflammation, representing another underrecognized pulmonary trade-off associated with conventional indoor housing without pharmacological growth promoters [70–72]. These findings emphasize the integration of structural and immune responses, as increased macrophage infiltration paralleled alveolar wall thickening and collagen accumulation, indicating chronic inflammatory remodeling [73, 74]. It should be noted, however, that macrophage identification in the present study was based solely on morphological criteria, and functional or phenotypic characterization (e.g., pro-inflammatory versus reparative macrophage states) was not performed.

Conversely, the free-range group exhibited the lowest macrophage focal density, indicating reduced inflammatory stress and more stable pulmonary immune homeostasis. Natural environmental exposure and increased physical activity may promote immune tolerance while limiting excessive inflammatory cell recruitment, reflecting organ-specific adaptation to enriched housing conditions. Although free-range animals encounter a broader range of environmental antigens, repeated low-level exposure may enhance mucosal immunity and tolerance, thereby reducing macrophage recruitment [75, 76]. In addition, larger alveolar spaces facilitate improved air exchange and inflammatory cell clearance [77]. The hygienic group showed intermediate macrophage densities, consistent with a balanced immune response under controlled biosecure conditions [78, 79]. Coordinated regulation of macrophage density in this group suggests that controlled environmental management can preserve pulmonary defense mechanisms without provoking excessive inflammatory remodeling [80].

Overall, histomorphometric and immunological findings demonstrate that management systems exert a substantial influence on lung microarchitecture, ECM remodeling, and immune regulation [81, 82]. Pulmonary structural and immune adaptation appears to function as an integrated system in which husbandry conditions co-modulate alveolar and bronchial remodeling, collagen deposition, and macrophage dynamics. Elevated macrophage density in beta-agonist-free animals reflects active immune engagement, whereas free-range swine show minimal inflammatory challenge and hygienic systems maintain immune equilibrium. Future studies incorporating immunophenotyping or molecular markers of macrophage activation are needed to further define macrophage functional states across management systems.

### Environmental and management considerations

Animals originating from different management systems were exposed to distinct production environments, and farm-level factors such as air quality, pathogen exposure, genetics, and management intensity may have contributed to the observed histological differences. A key limitation of this study is the absence of quantitative environmental exposure measurements, including ammonia concentration, airborne dust levels, and ventilation rates. Although temperature and humidity were controlled in beta-agonist-free and hygienic systems, these parameters could not be strictly regulated in free-range systems. Nevertheless, climatic conditions were broadly comparable across all groups because the study was conducted within the same geographic region of Thailand.

### Health and translational implications

Although the histological alterations identified in this study represent subclinical pulmonary remodeling below thresholds associated with overt respiratory disease, they may carry important One Health implications. Differences in lung structure, fibrosis, and macrophage density may reflect environmental exposures, such as dust, ammonia, and microbial load, that could also affect the respiratory health of farm workers. Future investigations incorporating systematic environmental monitoring and occupational exposure assessments may help establish clearer links among swine pulmonary health, human health, and environmental safety, thereby informing management strategies that optimize welfare and reduce cross-species risk.

### Future directions and reversibility of pulmonary remodeling

Future longitudinal studies are needed to clarify mechanistic links between housing environment and pulmonary remodeling and to identify early histological or molecular biomarkers of subclinical adaptation. The potential reversibility of fibrotic changes following modification of management conditions remains unexplored. Interventions such as improved ventilation, reduced stocking density, or environmental enrichment could be evaluated for their capacity to mitigate or reverse early interstitial fibrosis and restore alveolar architecture.

### Implications for swine production and welfare

Overall, these findings demonstrate that housing and management systems play a critical role in shaping pulmonary microarchitecture and immune regulation in swine. This study provides one of the first quantitative, lobe-specific comparisons of fibrosis and alveolar macrophage distribution across beta-agonist-free, hygienic, and free-range systems, revealing previously underexplored links between environmental management and lung structural adaptation. Management strategies that optimize biosecurity, ventilation, and opportunities for natural behavior may reduce inflammatory–fibrotic remodeling, preserve alveolar architecture, enhance gas exchange efficiency, and maintain pulmonary immune homeostasis, thereby improving animal welfare, reducing susceptibility to respiratory disease, and potentially enhancing production outcomes.

## CONCLUSION

**Figure 6:**
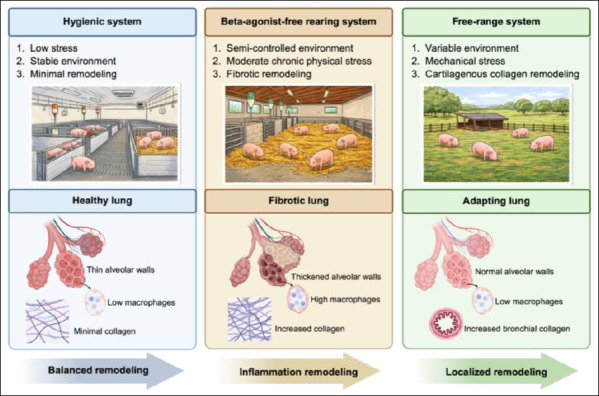
Conceptual schematic illustrating management system–driven pulmonary remodeling in pigs. The diagram summarizes how different swine management systems, including beta-agonist-free rearing, hygienic housing, and free-range systems, influence lung structural and immunological parameters.

This study demonstrates that management systems exert a clear and lobe-specific influence on pulmonary microarchitecture, ECM remodeling, and immune regulation in swine. Beta-agonist-free rearing was consistently associated with increased alveolar wall thickness, reduced alveolar space area, elevated PF indices, higher collagen deposition, and increased AMD, particularly in the middle and caudal lobes, indicating a restrictive and inflammatory–fibrotic remodeling pattern. In contrast, free-range management preserved alveolar structure, characterized by thinner septa, wider alveolar spaces, minimal PF, and the lowest AMD, reflecting enhanced ventilation efficiency and reduced inflammatory burden. The hygienic system showed intermediate features across most parameters, suggesting a balanced pulmonary phenotype under controlled housing with adequate ventilation. Together, these findings confirm that pulmonary adaptation is region-specific and strongly shaped by housing and management conditions (Figure 6).

From a production and welfare perspective, the results highlight that management systems designed to improve food safety or productivity may carry unintended pulmonary costs. Beta-agonist-free rearing, while advantageous for residue avoidance, may predispose animals to early subclinical pulmonary remodeling and immune activation under conventional indoor conditions. Free-range systems appear to support pulmonary resilience but may induce localized bronchial connective tissue adaptation in response to greater mechanical and environmental variability. Hygienic systems, when coupled with effective ventilation and environmental control, may offer a pragmatic compromise by maintaining pulmonary structural equilibrium. These insights support the refinement of housing, ventilation, and environmental management strategies to optimize respiratory health and overall welfare.

Key strengths of this study include the quantitative, lobe-specific histomorphometric approach; the integrated assessment of alveolar structure, PF, collagen intensity in BHC, and AMD; and the use of standardized, blinded analyses with sufficient statistical power to detect biologically meaningful differences. By focusing on the lung as both a functional and sentinel organ, this work extends beyond traditional performance metrics and provides organ-level evidence of management-related adaptation.

Several limitations should be acknowledged. Environmental exposures, such as ammonia concentration, airborne dust, and ventilation rates were not quantitatively measured, limiting direct attribution of histological changes to specific environmental stressors. PF assessment relied on Masson’s trichrome staining and semi-quantitative scoring without molecular or immunohistochemical confirmation of fibrotic pathways (e.g., collagen subtypes or TGF-β signaling). In addition, AMD was evaluated morphologically without functional or phenotypic characterization of macrophage subsets. The cross-sectional design also precludes conclusions regarding the temporal progression or reversibility of pulmonary remodeling.

Future studies should incorporate longitudinal designs to determine whether observed pulmonary changes are progressive or reversible following management modification. Integration of molecular, immunohisto-chemical, and immunophenotyping approaches will be essential to clarify mechanisms underlying PF and immune activation. Quantitative environmental monitoring and intervention trials targeting ventilation, stocking density, and enrichment would further strengthen causal inference and guide evidence-based management improve-ments.

In conclusion, this study provides robust evidence that housing and management systems differentially shape pulmonary structure and immune status in swine in a lobe-specific manner. Pulmonary adaptation emerges as an integrated structural–immunological response to environmental and management conditions. Optimizing housing design, ventilation, and environmental quality has the potential to mitigate subclinical inflammatory–fibrotic remodeling, preserve alveolar architecture, and support sustainable, welfare-oriented swine production.

## DATA AVAILABILITY

All data generated or analyzed during this study are included in this article. Raw histological images and quantitative datasets used to support this study’s findings are available from the corresponding author upon reasonable request.

## AUTHORS’ CONTRIBUTIONS

NC: Analyzed the data and drafted and revised the manuscript. NP and SC: Conducted laboratory experiments and collected and analyzed data. PJ and IP: Contributed to study design and data interpretation. CP: Conceptualized and designed the study, interpreted data, and critically drafted and revised the manuscript. All authors have read and approved the final version of the manuscript.
